# Lenvatinib with or without stereotactic body radiotherapy for hepatocellular carcinoma with portal vein tumor thrombosis: a retrospective study

**DOI:** 10.1186/s13014-023-02270-z

**Published:** 2023-06-12

**Authors:** Xiaoquan Ji, Zhe Xu, Jing Sun, Wengang Li, Xuezhang Duan, Quan Wang

**Affiliations:** 1 Department of Radiation Oncology, Senior Department of Oncology, The Fifth Medical Center of PLA General Hospital, Beijing, China; 2 The Second School of Clinical Medicine, Southern Medical University, Guangzhou, China; 3 Treatment and Research Center for Infectious Diseases, The Fifth Medical Center of PLA General Hospital, National Clinical Research Center for Infectious Diseases, Beijing, 100039 China

**Keywords:** Hepatocellular carcinoma, Portal vein tumor thrombosis, Stereotactic body radiotherapy, Lenvatinib

## Abstract

**Background and objectives:**

Patients with hepatocellular carcinoma (HCC) involving portal vein tumor thrombosis (PVTT) are presently lacking effective treatment options. We aimed to compare the efficacy and safety of lenvatinib with or without SBRT for HCC with PVTT.

**Materials and methods:**

This retrospective analysis included 37 patients treated with lenvatinib in combination with SBRT and 77 patients treated with lenvatinib alone from August 2018 to August 2021. Overall survival (OS), progression-free survival (PFS), intrahepatic PFS (IHPFS) and objective remission rate (ORR) were compared between the two groups, while adverse events (AEs) was analyzed between the two groups to assess safety profiles.

**Results:**

Median OS, PFS and IHPFS were significantly prolonged in the combination treatment group compared with the single treatment group (median OS, 19.3 vs. 11.2 months, *p* < 0.001; median PFS: 10.3 vs. 5.3 months, *p* < 0.001; median IHPFS, 10.7 vs. 5.3 months, *p* < 0.001). Moreover, a higher ORR (56.8% vs. 20.8%, *P* < 0.001) were observed in the lenvatinib combined with SBRT group. In subgroup analyses of Vp1-2 and Vp3-4 group, median OS, PFS and IHPFS were also significantly longer in the lenvatinib combined with SBRT group than those in the lenvatinib alone group. AEs in the combined therapy group were mostly manageable and the incidence was not statistically significant compared to the monotherapy group.

**Conclusion:**

Lenvatinib plus SBRT had a significantly better survival benefit than lenvatinib monotherapy in the treatment of HCC patients with PVTT and was well tolerated.

**Supplementary Information:**

The online version contains supplementary material available at 10.1186/s13014-023-02270-z.

## Introduction

Hepatocellular carcinoma (HCC) is the leading cause of cancer-related death and the incidence is predicted to continue to rise [1, 2]. Due to liver anatomy and HCC biology, HCC is likely to invade the adjacent vasculature. Portal vein tumor thrombosis (PVTT) is the predominant form of macrovascular invasion, with a prevalence of 44–62% at autopsy [3, 4]. HCC with PVTT was considered to be advanced stage [5] and had an extremely poor prognosis, with an overall survival (OS) as low as 2.7–4 months if receiving supportive care treatment only [3].

The multi-tyrosine kinase inhibitors (TKIs) are the recommended as first-line treatments for advanced HCC with PVTT [6, 7]. Sorafenib is the only first-line systemic therapy proven to prolong OS compared to placebo until the introduction of Lenvatinib in 2018 [8]. A phase III REFLECT trial showed that lenvatinib was the first drug with non-inferiority to sorafenib in OS [9]. Hence, lenvatinib was established as an alternative to sorafenib as first-line treatment in many countries (including China) [10]. Nevertheless, the efficacy of these TKI-targeted drugs still remained suboptimal, and alternative strategies that can improve outcomes are still urgently needed.

With advances in radiotherapy technology, external beam radiotherapy such as stereotactic body radiation therapy (SBRT) has been an effective and safe alternative for HCC patients [11]. A meta-analysis showed that SBRT had an effective rate of 70.7% in the treatment of PVTT, which was much higher than that of other radiotherapy methods [12]. Moreover, SBRT, alone or in combination with systemic therapy, can be used also for the treatment of liver metastasis [13]. Local treatment such as SBRT can rapidly reduce intrahepatic tumor load and enhance the antitumor effects of TKI-targeted drugs in patients with advanced HCC [14]. Furthermore, previous studies have shown that sorafenib selectively inhibits activation of vascular endothelial growth factor receptor 2 (VEGFR2) and its downstream signaling pathway induced by radiotherapy, and increases radiation-induced apoptosis [15]. Based on these theories, SBRT combined with TKI could be a very promising combination [16, 17].

The combination of radiotherapy and sorafenib has not been widely used in clinical practice because of uncertain clinical efficacy [18]. Compared with sorafenib, lenvatinib showed statistically significant improvement in objective response rate (ORR), progression-free survival (PFS) and time to progression (TTP) in a phase III clinical, moreover, lenvatinib had a stronger inhibitory effect on the VEGF and fibroblast growth factor receptor signaling [19]. However, there is currently a lack of comparative studies on the efficacy of radiotherapy combined with lenvatinib versus lenvatinib alone for hepatocellular carcinoma with PVTT. Therefore, we conducted this retrospective cohort study to compare the efficacy and safety of lenvatinib plus SBRT versus lenvatinib monotherapy as first-line treatment in Chinese patients with advanced HCC involving PVTT.

## Materials and methods

### Study design and patient enrollment

Patients with unresectable HCC diagnosed from August 2018 to August 2021 were included in this retrospective study. The eligibility criteria were: (1) histologic or clinical diagnosis of HCC, (2) Treated with Lenvatinib, (3) presence of PVTT and PVTT was identified by the existence of a low attenuation intraluminal filling defect during the portal phase and a filling defect enhancement during the arterial phase, (4) at least one measurable lesion ≥ 1 cm in solid liver lesion or vascular tumor thrombosis > 1 cm, (5) Child–Pugh classification A and B, (6) Eastern Cooperative Oncology Group performance status score (ECOG PS) 0–1. Exclusion criteria were as follows: (1) concomitant with other malignancy, (2) presence of extrahepatic metastases, (3) previously received any systemic therapy, (4) combination with other treatments including TACE, radiofrequency ablation and immune checkpoint inhibitors, (5) lack of baseline radiological imaging, and (6) lost to follow-up. We classified PVTT into five types based on the type of PVTT classification proposed by the Japanese Hepatocellular Carcinoma Research Group [20], based on the severity of tumor thrombosis and anatomical structure: Vp0, no PVTT; Vp1, PVTT distal to but not involved in second-order branches of the PV; Vp2, PVTT invasive to second-order branches; Vp3, PVTT present in first-order branches; Vp4, PVTT extends into the main portal trunk and/or contralateral portal vein branches. This study was approved by the Ethics Committee of the Fifth Medical Center of the General Hospital of the Chinese People's Liberation Army (procedure code 2020 - 063 - D) and the requirement for informed consent was waived due to the retrospective study design.

### SBRT therapy

After implanting 3–5 fiducial markers in each patient, they received CyberKnife®-SBRT (CK-SBRT) using a CyberKnife® VSI image-guided robotic stereotactic radiosurgery system (Accuray inc., Sunnyvale, CA, USA). After locating the treatment location using computed tomography (CT) simulation, an oncologist contoured the gross tumor volume (GTV) and outlined organs at risk (OARs). The GTV was conventionally defined as the total volume of PVTT and parenchymal HCC. However, in patients with large tumors and severe liver cirrhosis or numerous intrahepatic metastases, only the PVTT was delineated as the GTV. The planning target volume (PTV) expanded 3–5 mm of the GTV and avoided the OARs. The prescribed doses were 45–55 Gy/5–10 fx. The plans were calculated using CyberKnife® Multiplan® Treatment Planning System software (version 4.0.2), and the tolerance doses of OARs were determined based on the American Association of Physicists in Medicine (AAPM) TG-101 report [21].

### Lenvatinib therapy

All patients started oral lenvatinib treatment within 7 days after completion of the last SBRT. For patients with Child–Pugh classification A, the regular starting dose of lenvatinib is 12 mg/d for patients weighing > 60 kg and 8 mg/d for patients weighing ≤ 60 kg. For patients with Child–Pugh classification limited B, the regular starting dose is 8 mg/d, regardless of weight [22, 23]. If participants experience ≥ Grade 3 or unacceptable drug-related AEs, adjust or interrupt the lenvatinib dose according to the manufacturer's instructions until the AE is reduced to Grade 1 or disappears.

### Follow-up study

The time of first follow-up was 4–8 weeks after treatment and every 2–3 months thereafter until Sep 30, 2022, or when the patient died. Follow-up tests included blood routine, liver function, coagulation function, serum tumor markers, contrast-enhanced CT or MRI of the upper abdomen, and lung CT. OS was defined as the time from the date of initiation of SBRT to the date of death from any cause or the date of last follow-up (Sep 30, 2022). PFS was defined as the time from the date of initiation of SBRT to the date of first detection of tumor progression or death from any cause, based on the Modified Response Evaluation Criteria in Solid Tumors (mRECIST) [24, 25]. IHPFS was defined as the time from the date of initiation of SBRT to the date of first detection of intrahepatic tumor progression or death from any cause.

### Response evaluation and toxicity reaction evaluation

Local tumor response was assessed using mRECIST based on images obtained at the first time of follow-up, and were classified as complete response (CR), partial response (PR), stable disease (SD), and progressive disease (PD). ORR = CR + PR, and disease control rate (DCR) = CR + PR + SD. The toxicity reaction was evaluated according to the Common Terminology Criteria for Adverse Events (CTCAE) version 5.0 [26].

### Statistical analysis

To compare the differences between groups, an independent t-test was used for numerical variables, and chi-square test or Fisher’s exact test was used for categorical variables. Survival rates were estimated by the Kaplan–Meier method. Univariate and multivariate Cox proportional hazards models were applied to survival outcomes for OS, PFS and IHPFS. Variables with *p* values < 0.1 in the univariate model were included in the multivariate model. Risk ratios and 95% confidence intervals were calculated to determine statistical significance. *P* value < 0.05 was considered statistically significant. All statistical analyses were performed using SPSS ver. 26.0 (IBM Corp., Armonk, NY, USA).

## Results

### Patient characteristics

**Fig. 1:**
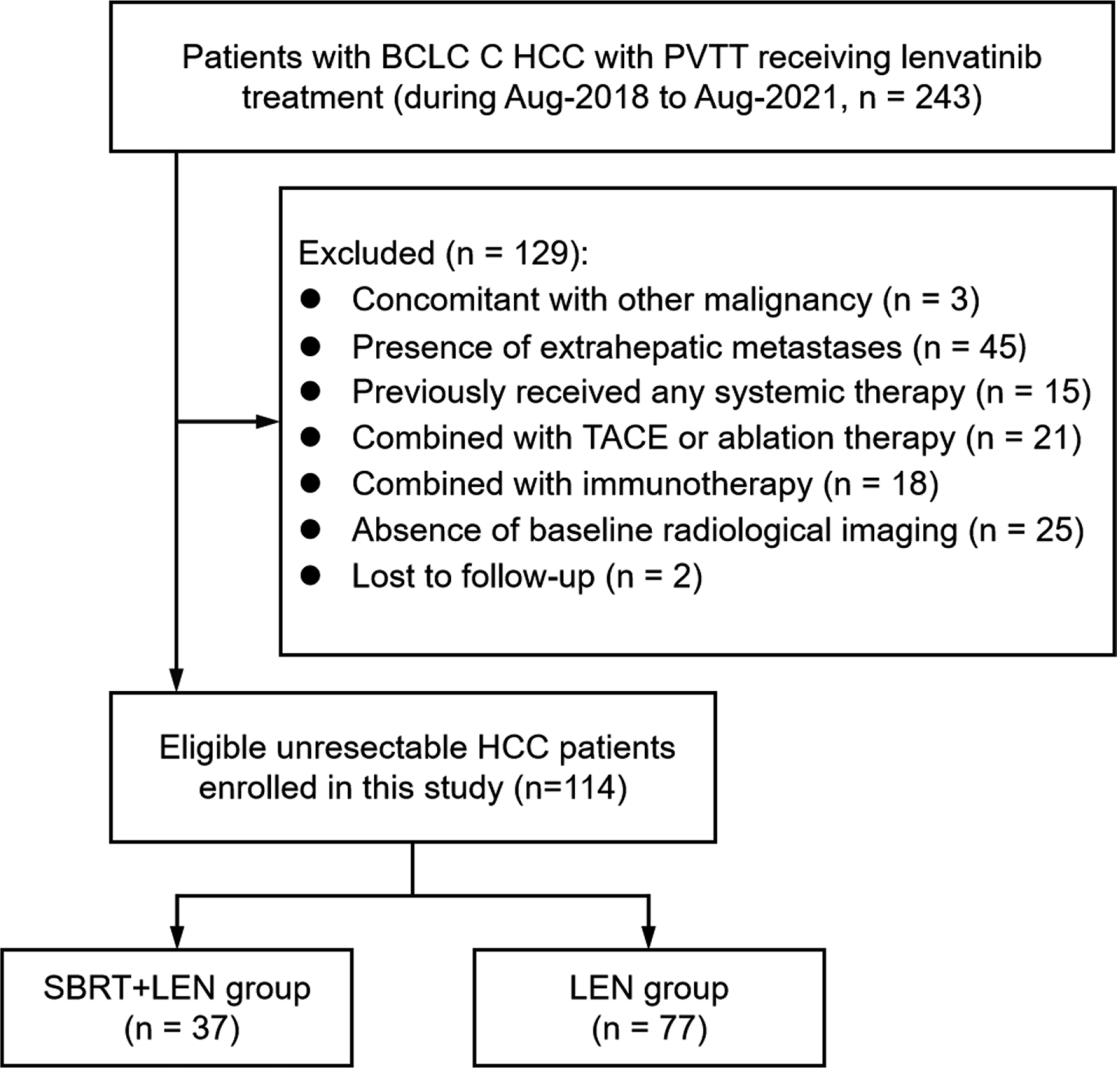
Patient flowchart. TACE, transarterial chemoembolization; SBRT, stereotactic body radiotherapy; LEN, lenvatinib

A total of 246 patients with unresectable HCC treated with lenvatinib were retrospectively reviewed, of them 132 patients did not meet the inclusion criteria. Of the remaining 114 patients, 37 patients received a combination of SBRT and Lenvatinib (SBRT + LEN group) and 77 received lenvatinib alone (LEN group) (Fig. 1). The baseline characteristics of two groups are summarized in Table 1, and all of the baseline characteristics were well-balanced between the two groups. According to the degree of PVTT, the whole cohort of 114 patients were divided into two subgroups (Vp1-2 group and Vp3-4 group). The baseline characteristics of Vp1-2 group and Vp3-4 group were also well-balanced (Additional file 1: Table S1). It is noteworthy that approximately 90% of patients did not receive any previous treatment, and 44.7% (51/114) of the population in our study were out of the REFLECT criteria, with extensive tumor burden and poor liver function and performance status.

**Table 1:** Baseline characteristics of the study population

Characteristics	Total enrolled patients (n = 114)	SBRT + LEN group (n = 37)	LEN group (n = 77)	*p* value
Gender, n (%)				
Male	100 (87.8%)	32 (86.5%)	68 (88.3%)	1.000
Female	14 (12.2%)	5 (13.5%)	9 (11.7%)	
Age, years, n (%)				
≥ 55 years	60 (52.7%)	21 (56.8%)	39 (50.6%)	0.541
< 55 years	54 (47.3%)	16 (43.2%)	38 (49.4%)	
HBV infection, n (%)				
Yes	107 (93.9%)	34 (91.9%)	73 (94.8%)	0.680
No	7 (6.1%)	3 (8.1%)	4 (5.2%)	
Child–Pugh classification, n (%)				
A	96 (84.2%)	31 (83.8%)	65 (84.4%)	0.931
B	18 (15.8%)	6 (16.2%%)	12 (15.6%)	
ECOG PS score, n (%)				
0	49 (42.9%)	17 (45.9%)	32 (41.6%)	0.658
1	65 (57.1%)	20 (54.1%)	45 (58.4%)	
Number of tumors, n (%)				
> 3	57 (50.0%)	17 (45.9%)	40 (51.9%)	0.548
≤ 3	57 (50.0%)	20 (54.1%)	37 (48.1%)	
Tumor size, n (%)				
< 5 cm	23 (20.2%)	7 (18.9%)	16 (20.8%)	0.765
≥ 5 cm and < 10 cm	53 (46.5%)	19 (51.4%)	34 (44.1%)	
≥ 10 cm	38 (33.3%)	11 (29.7%)	27 (35.1%)	
PVTT, n (%)				
VP1	7 (6.1%)	1 (2.7%)	6 (7.8%)	0.707
VP2	44 (36.9%)	16 (43.2%)	28 (36.4%)	
VP3	53 (50.9%)	17 (45.9%)	36 (46.8%)	
VP4	10 (6.1%)	3 (8.1%)	7 (9.1%)	
ALBI grade, n (%)				
1	34 (29.8%)	13 (35.1%)	21 (27.3%)	0.390
2	80 (70.2%)	24 (64.9%)	56 (72.7%)	
AFP, n (%)				
> 200 ng/mL	55 (48.2%)	18 (48.6%)	37 (48.1%)	0.952
≤ 200 ng/mL	59 (51.8%)	19 (51.4%)	40 (51.9%)	
PLT, n (%)				
≥ 100 × 10^9^/L	86 (75.5%)	28 (75.7%)	58 (75.3%)	0.967
< 100 × 10^9^/L	28 (24.5%)	9 (24.3%)	19 (24.7%)	
WBC, n (%)				
≥ 4 × 10^9^/L	78 (68.5%)	25 (67.6%)	53 (68.8%)	0.892
< 4 × 10^9^/L	36 (31.5%)	12 (32.4%)	24 (31.2%)	
Previous local treatment, n (%)				
Absence	102 (89.5%)	34 (91.9%)	68 (88.3%)	0.797
Presence				
TACE	5 (4.4%)	2 (5.4%)	3 (3.9%)	
Ablation	3 (2.6%)	1 (2.7%)	2 (2.6%)	
Argon–Helium cryosurgical	4 (3.5%)	0 (0%)	4 (5.2%)	

*HBV* hepatitis b virus, *ECOG* Eastern Cooperative Oncology Group, *PS* performance status, *PVTT* Portal Vein Tumor Thrombosis, *ALBI* grade albumin-bilirubin grade, *AFP* alpha-fetoprotein, *PLT* platelet, *WBC* white blood cell, *TACE* transarterial chemoembolization, *SBRT* stereotactic body radiotherapy, *LEN* lenvatinib

### Treatment efficacy in the whole cohort

**Fig. 2:**
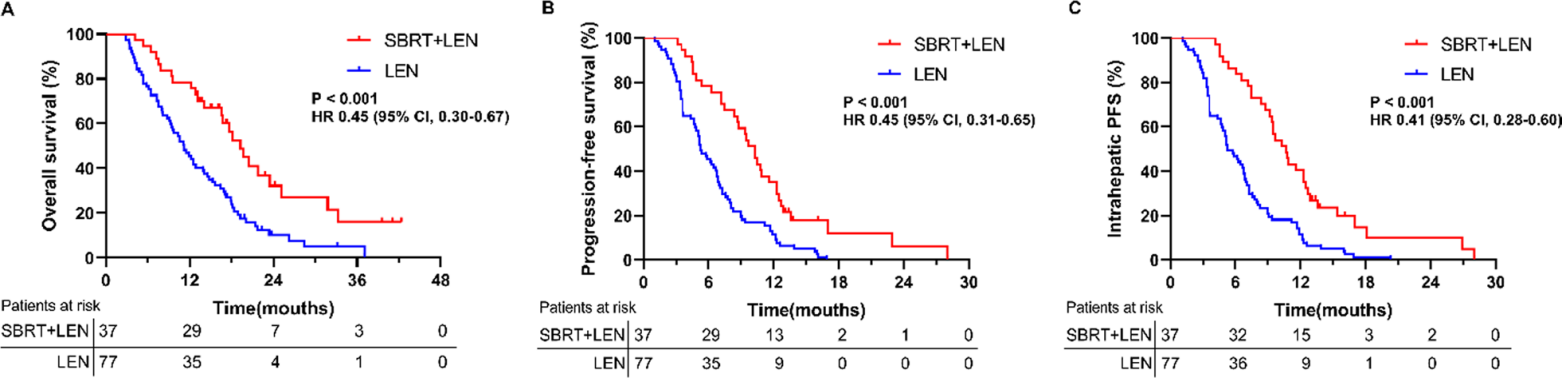
Overall survival (OS), progression-free survival (PFS) and intrahepatic PFS (IHPFS) in different groups. **A** The OS in the total cohort. **B** The PFS in the total cohort. **C** The IHPFS in the total cohort

The median follow-up durations for the SBRT plus lenvatinib and lenvatinib groups were 39.6 [95% CI 18.7, 60.6] and 33.2 [95% CI 22.3, 44.1] months, respectively. The patients in the SBRT plus lenvatinib group had longer OS (median, 19.3 [95% CI 16.0, 22.6] months) than those in the lenvatinib group (median, 11.2 [95% CI 8.8, 13.6] months; [HR] 0.45, [95% CI 0.30, 0.67], *P* < 0.001; Fig. 2A). The median PFS in the SBRT plus lenvatinib group (10.3 [95% CI 9.0, 11.6] months) was longer than in the lenvatinib group (5.3 [95% CI 4.2, 6.4] months) (HR 0.45, [95% CI 0.31, 0.65], *p* < 0.001; Fig. 2B). A longer median IHPFS (10.7 [95% CI 9.0, 12.4] vs. 5.3 [95% CI 4.2, 4.7] months) was also observed in patients treated with the combined therapy compared with the monotherapy (HR 0.41, [95% CI 0.28, 0.60], *p* < 0.001; Fig. 2C).

**Fig. 3:**
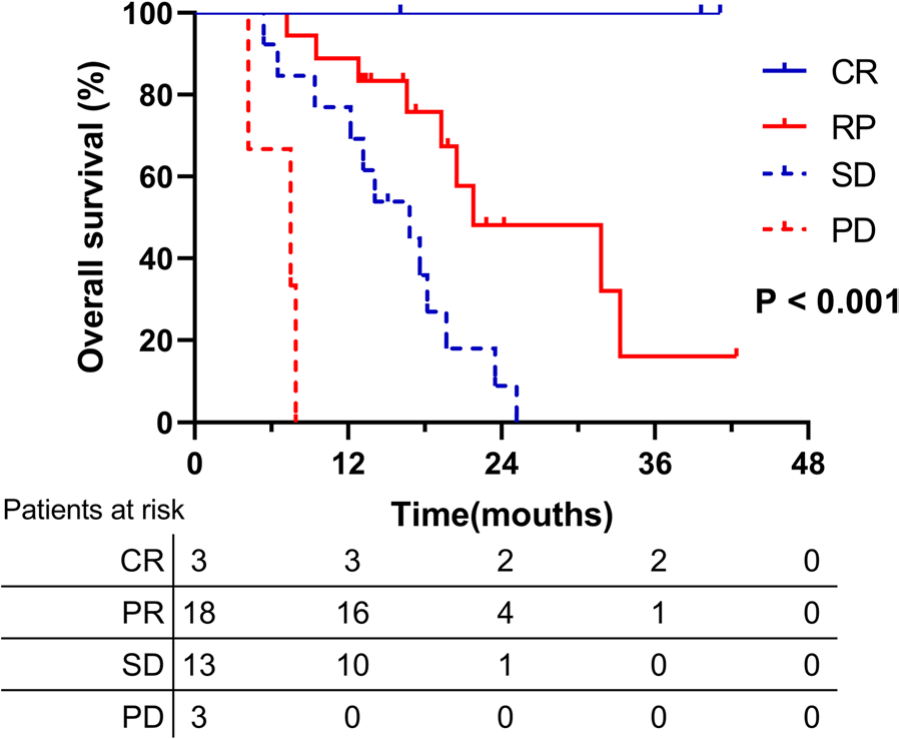
The overall survival in the SBRT + LEN group according to treatment response

The best tumor response evaluated by mRECIST criteria are shown in Table 2. The ORR in the SBRT plus lenvatinib group was 56.8%, significantly higher than the 20.8% ORR observed in the lenvatinib group (*p* < 0.001). In addition, 34 cases in the SBRT plus lenvatinib group vs. 50 cases in the lenvatinib group achieved disease control (DCR: 91.9% vs. 64.9%, *p* = 0.005). Furthermore, patients who showed treatment response in the SBRT plus lenvatinib group had significantly higher OS than those who did not respond to treatment (*p* < 0.001) (Fig. 3).

**Table 2:** Best tumor response evaluated by mRECIST

mRECIST	Total enrolled Patients (n = 114)	SBRT + LEN group (n = 37)	LEN group (n = 77)	*P*^a^ value
Complete response, n (%)	4 (3.5%)	3 (8.1%)	1 (1.3%)	0.100
Partial response, n (%)	32 (28.1%)	18 (46.8%)	15 (19.5%)	**0.001**
Stable disease, n (%)	48 (42.1%)	13 (35.1%)	34 (44.2%)	0.360
Progressive disease, n (%)	30 (26.3%)	3 (8.1%)	27 (35.1%)	**0.005**
ORR, n (%)	36 (31.6%)	21 (56.8%)	16 (20.8%)	**0.000**
DCR, n (%)	84 (73.7%)	34 (91.9%)	50 (64.9%)	**0.005**

*mRECIST* modified Response Evaluation Criteria in Solid Tumors, *ORR* objective response rate = complete response rate + partial response rate

^a^Bold values indicate statistical significance

### Treatment efficacy in the subgroup

**Fig. 4:**
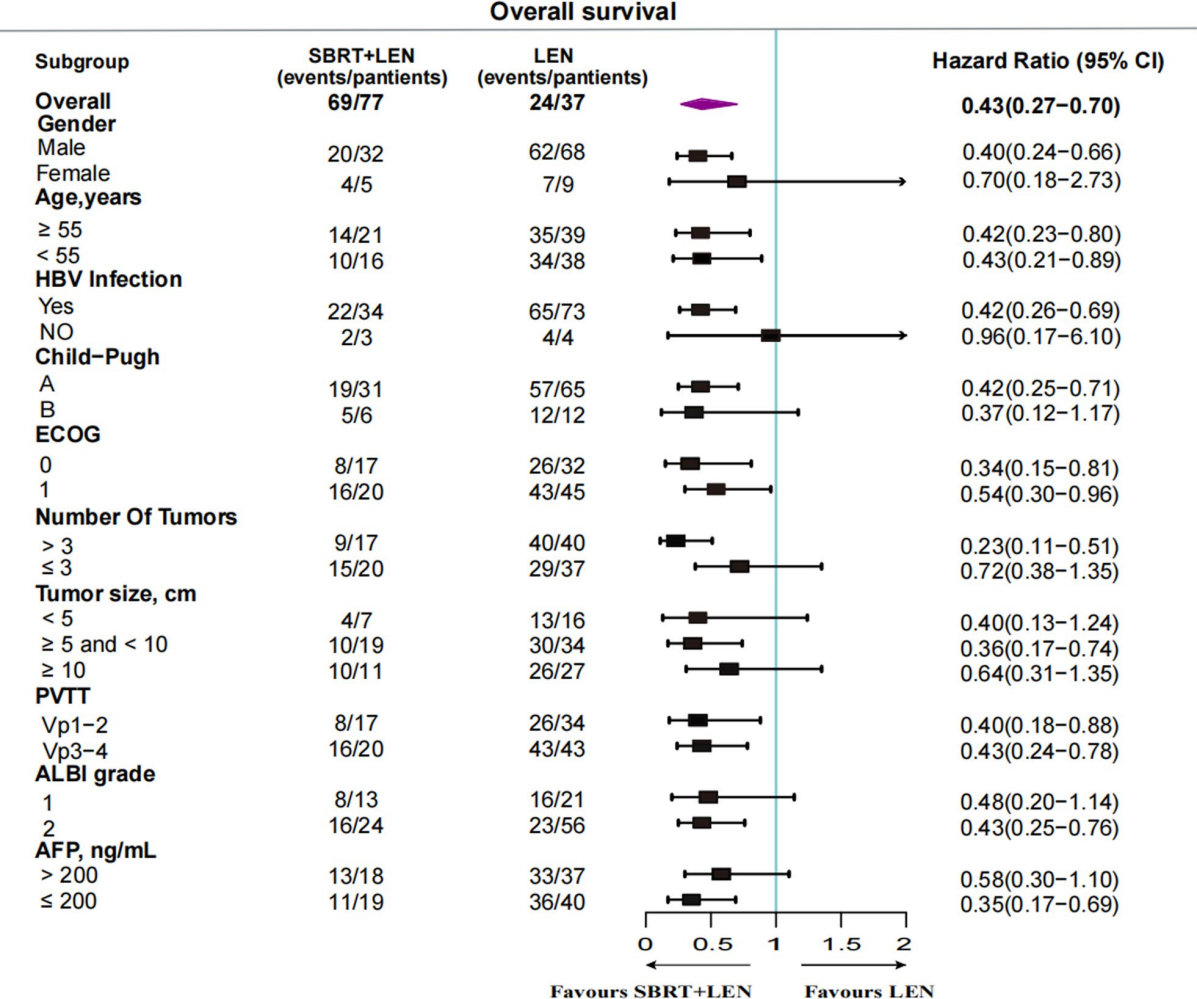
Subgroup analyses of overall survival in the patients. HBV, hepatitis b virus, ECOG = Eastern Cooperative Oncology Group, PVTT = Portal Vein Tumor Thrombosis, ALBI = albumin-bilirubin, AFP = a-fetoprotein, SBRT = stereotactic body radiotherapy, LEN = lenvatinib

Forest plot analysis of OS related factors showed that the benefit of SBRT combined with lenvatinib exceeded that of lenvatinib monotherapy in the patients of male, age ≥ 55 or < 55, HBV infection, Child–Pugh class A, ECOG score 0 or 1, number of tumors > 3, tumor size ≥ 5 cm and < 10 cm, PVTT Vp1-2 or Vp3-4, ALBI grade 2, and AFP ≤ 200 (Fig. 4).

**Fig. 5:**
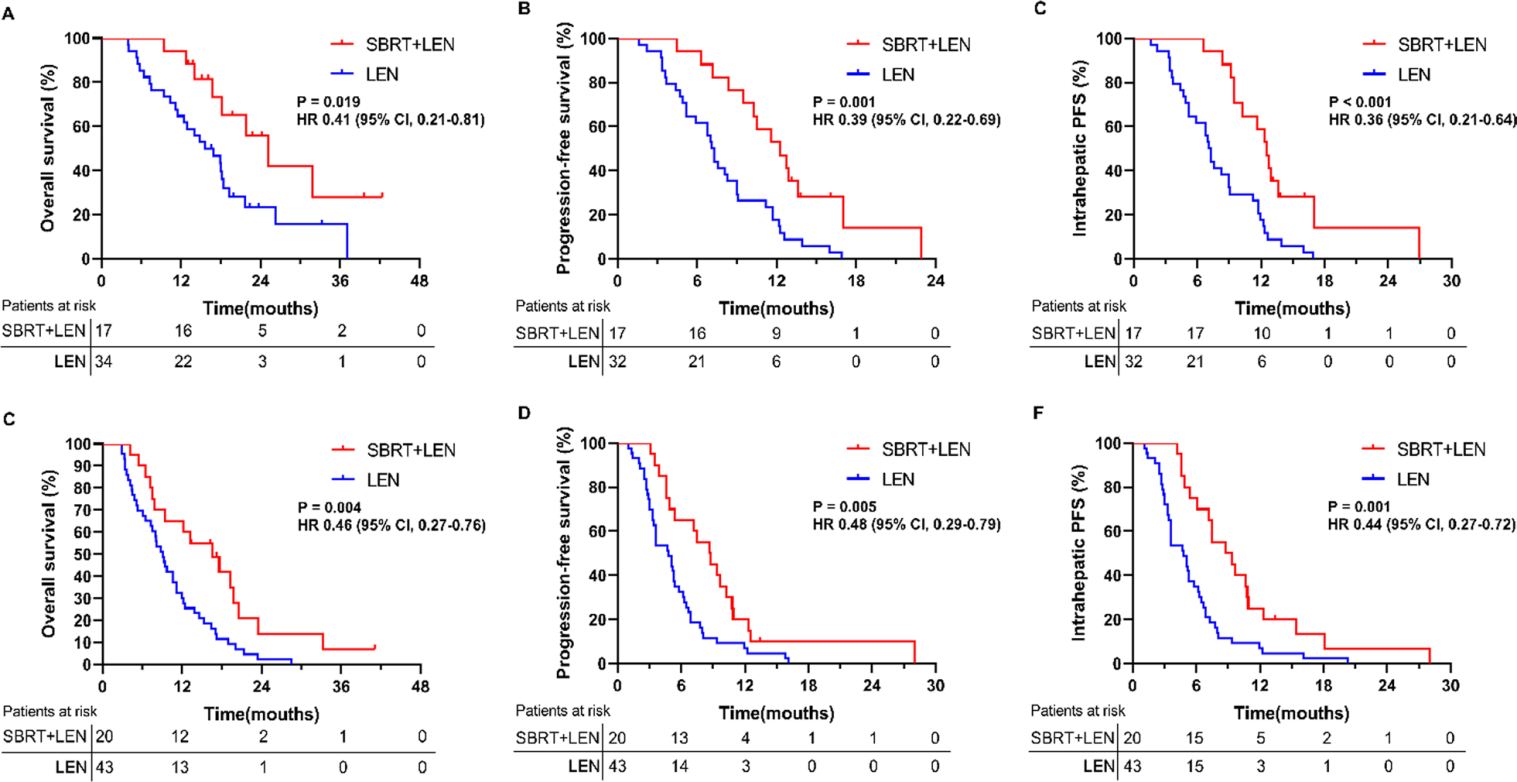
Overall survival (OS), progression-free survival (PFS) and intrahepatic PFS (IHPFS) in the subgroups. **A** The OS in the Vp1-2 subgroup. **B** The PFS in the Vp1-2 subgroup. **C** The IHPFS in Vp1-2 subgroup. **D** The OS in the Vp3-4 subgroup. **E** The PFS in the Vp3-4 subgroup. **F** The IHPFS in Vp3-4 subgroup

In the subgroup analysis of Vp1-2 group, the median OS (25.2 [95% CI 17.6, 32.8] vs. 15.7 [95% CI 10.6, 20.8] months; [HR] 0.41, [95% CI 0.21, 0.81], *P* = 0.019; Fig. 5A), PFS (12.3 [95% CI 19.3, 15.3] vs. 7.1 [95% CI 6.3, 7.8] months; [HR] 0.39, [95% CI 0.22, 0.69], *P* = 0.001; Fig. 5B) and IHPFS (12.5 [95% CI 11.0, 14.0] vs. 7.1 [95% CI 6.3, 7.8] months; [HR] 0.36, [95% CI 0.21, 0.64], *P* < 0.001; Fig. 5C) in SBRT plus lenvatinib group was significantly longer than in lenvatinib group. In the Vp3-4 group, a longer median OS (16.6 [95% CI 9.1, 24.1] vs. 9.1 [95% CI 7.4, 10.8] months; [HR] 0.46, [95% CI 0.27, 0.76], *P* = 0.004; Fig. 5D), PFS (8.7 [95% CI 5.9, 11.5] vs. 4.8 [95% CI 2.9, 6.8] months; [HR] 0.48, [95% CI 0.29, 0.79], *P* = 0.005; Fig. 5E) and IHPFS (8.8 [95% CI 4.6, 13.0] vs. 4.8 [95% CI 3.4, 6.2] months; [HR] 0.44, [95% CI 0.27, 0.72], *P* = 0.001; Fig. 5F) were also observed in the combined therapy group.

### Prognostic factors for OS, PFS and IHPFS in the whole cohort

The independent prognostic factors associated with OS, PFS and IHPFS were performed Univariate and multifactorial analyses based on Cox regression models (Table 3). Multivariate Cox proportional risk analysis showed that SBRT plus lenvatinib significantly improved OS ([HR] 0.37; [95% CI 0.26, 0.60]; *P* = 0.001), PFS ([HR] 0.33; [95% CI 0.21–0.51]; *P* < 0.001), and IHPFS ([HR] 0.29; [95% CI 0.18–0.45]; *P* < 0.001). Besides, the number of tumors and the degree of PVTT were also independent risk factors for OS, PFS and IHPFS.

**Table 3:** Univariate and multivariate Cox regression analysis on OS, PFS and IHPFS in the whole cohort

Variable	OS (N = 114, events = 91)				PFS (N = 114, events = 110)				IHPFS (N = 114, events = 110)			
	Univariable model		Multivariable model		Univariable model		Multivariable model		Univariable model		Multivariable model	
	HR (95% CI)	*P*^a^	HR (95% CI)	*p*^a^	HR (95% CI)	*p*^a^	HR (95% CI)	*p*^a^	HR (95% CI)	*p*^a^	HR (95% CI)	*p*^a^
Group (refer to LEN)	0.43 (0.27–0.70)	**0.001**	0.37 (0.23–0.60)	**0.000**	0.41 (0.27–0.63)	**0.000**	0.33 (0.21–0.51)	**0.000**	0.38 (0.25–0.59)	**0.000**	0.29 (0.18–0.45)	**0.000**
Gender (refer to Male)	1.21 (0.65–2.28)	0.548			1.53 (0.87–2.70)	0.145			1.28 (0.73–2.25)	0.390		
Age (refer to < 55 years)	1.01 (0.67–1.52)	0.960			1.11 (0.76–1.62)	0.605			1.10 (0.74–1.60)	0.668		
Etiology (refer to HBV)	1.49 (0.64–3.45)	0.356			0.93 (0.41–2.12)	0.864			0.96 (0.42–2.20)	0.924		
Child–Pugh classification (refer to A)	1.87 (1.09–3.23)	**0.024**	1.15 (0.63–2.11)	0.655	1.57 (0.93–2.65)	0.089	1.28 (0.74–2.20)	0.382	1.68 (1.00–2.84)	0.052	1.38 (0.80–2.38)	0.248
ECOG PS (refer to 0)	1.71 (1.12–2.62)	**0.013**	1.37 (0.85–2.20)	0.199	1.35 (0.92–1.98)	0.127			1.34 (0.91–1.97)	0.135		
Number of tumors (refer to ≤ 3)	1.79 (1.18–2.71)	**0.006**	1.73 (1.11–2.72)	**0.017**	1.83 (1.25–2.68)	**0.002**	2.05 (1.36–3.09)	**0.001**	1.77 (1.21–2.59)	**0.004**	2.05 (1.35–3.12)	**0.001**
Tumor size (refer to < 5 cm)	1.21 (0.92–1.61)	0.180			1.11 (0.86–1.44)	0.429			1.10 (0.84–1.42)	0.491		
PVTT (refer to Vp1)	2.16 (1.56–2.99)	**0.000**	1.95 (1.37–2.77)	**0.000**	1.50 (1.14–1.97)	**0.004**	1.52 (1.13–2.04)	**0.005**	1.46 (1.11–1.92)	**0.008**	1.45 (1.08–1.95)	**0.014**
ALBI grade (refer to 1)	1.40 (0.88–2.23)	0.155			1.10 (0.73–1.67)	0.641			1.07 (0.71–1.62)	0.742		
AFP (refer t ≤ 200 ng/mL)	1.54 (1.02–2.32)	**0.039**	1.24 (0.81–1.89)	0.317	1.57 (1.07–2.31)	**0.021**	1.39 (0.94–2.05)	0.097	1.51 (1.03–2.21)	**0.036**	1.37 (0.92–2.02)	0.119
PLT (refer to < 100 × 109/L)	0.81 (0.51–1.31)	0.396			0.91 (0.59–1.42)	0.684			0.93 (0.59–1.44)	0.732		
WBC (refer to < 4 × 109/L)	0.84 (0.54–1.30)	0.429			0.69 (0.46–1.04)	0.078	0.67 (0.43–1.02)	0.063	0.68 (0.45–1.03)	0.072	0.66 (0.43–1.02)	0.063
Previous local treatment (refer to Absence)	0.77 (0.39–1.53)	0.454			0.95 (0.52–1.74)	0.870			0.86 (0.47–1.57)	0.623		

*HBV* hepatitis b virus, *ECOG* Eastern Cooperative Oncology Group, *PS* performance status, *MVI* macrovascular invasion, *ALBI* grade albumin-bilirubin grade, *AFP* alpha-fetoprotein, *PLT* platelet, *WBC* white blood cell, *LEN* lenvatinib, *TAE* transcatheter arterial embolization, *SBRT* stereotactic body radiotherapy, *LEN* lenvatinib, *PFS* progression-free survival, *OS* overall survival, *IHPFS* intrahepatic progression-free survival

^a^Bold values indicate statistical significance

### Treatment-related adverse events

The incidence of AEs at any grade in both groups was shown in Table 4. The overall incidence of at least 1 AE of any grade was similar in the SBRT plus lenvatinib and lenvatinib group (34 [91.9%] vs*.* 69 [89.6%], *P* = 0.962). In the SBRT plus lenvatinib and lenvatinib groups, dose reduction, dose interruption or discontinuation of lenvatinib treatment due to grade ≥ 3 AEs could be observed in 7 (18.9%) and 12 (15.6%) patients, respectively. The most common AEs in the combined therapy group were hypertension (10 [27.0%]) and diarrhea (10 [27.0%]), while in the monotherapy group the most common AEs were hypertension (24 [31.2%]) and fatigue (23 [29.9%]). Overall, AEs in the combined therapy group were mostly manageable and the incidence was not statistically significant compared to the monotherapy group.

**Table 4:** Treatment-related adverse events

Adverse events	All grades			Grade ≥ 3		
	Group		*P* value	Group		*P* value
	SBRT + LEN (n = 37)	LEN (n = 77)		SBRT + LEN (n = 37)	LEN (n = 77)	
Any adverse event, n (%)	34 (91.9%)	69 (89.6%)	0.962	7 (18.9%)	12 (15.6%)	0.655
Hypertension, n (%)	10 (27.0%)	24 (31.2%)	0.651	2 (5.4%)	6 (7.8%)	0.940
Nausea/vomiting, n (%)	8 (21.6%)	21 (27.3%)	0.517	1 (2.7%)	3 (3.9%)	1.000
Proteinuria, n (%)	6 (16.2%)	16 (20.8%)	0.563	0 (0%)	0 (0%)	–
Peripheral edema, n (%)	1 (2.7%)	3 (3.9%)	1.000	0 (0%)	0 (0%)	–
Fatigue, n (%)	9 (24.3%)	23 (29.9%)	0.537	0 (0%)	0 (0%)	–
Anorexia, n (%)	9 (24.3%)	16 (20.8%)	0.668	1 (2.7%)	0 (0%)	0.330
Abdominal pain, n (%)	8 (21.6%)	15 (19.5%)	0.928	1 (2.7%)	1 (1.3%)	0.546
Constipation, n (%)	3 (8.1%)	8 (10.4%)	0.962	0 (0%)	0 (0%)	1.000
Diarrhea, n (%)	10 (27.0%)	18 (23.4%)	0.672	1 (2.7%)	1 (1.3%)	0.546
Rash, n (%)	1 (2.7%)	6 (7.8%)	0.520	0 (0%)	0 (0%)	–
Hand-foot syndrome, n (%)	1 (2.7%)	8 (10.4%)	0.292	0 (0%)	1 (1.3%)	1.000
AST/ALT elevation, n (%)	8 (21.6%)	13 (16.9%)	0.787	1 (2.7%)	0 (0%)	0.330
Thrombocytopenia, n (%)	6 (16.2%)	6 (7.8%)	0.170	0 (0%)	0 (0%)	–
Leukopenia, n (%)	5 (13.5%)	5 (6.5%)	0.290	0 (0%)	0 (0%)	–
Fever, n (%)	1 (2.7%)	4 (5.2%)	0.543	0 (0%)	0 (0%)	–
Joint pain, n (%)	0 (0%)	4 (5.2%)	0.302	0 (0%)	0 (0%)	–

*AST* aspartate aminotransferase, *ALT* alanine aminotransferase, *SBRT* stereotactic body radiotherapy, *LEN* lenvatinib

### Subsequent therapy

During the follow-up period, all patients in lenvatinib group showed tumor progres-sion compared to 33 (89.2%) patients in the combination therapy group. 20 (60.6%) of 33 progressed patients in the SBRT plus lenvatinib group received subsequent therapy, compared with 45 (58.4%) of 77 progressed patients in the lenvatinib group. Among the patients who received subsequent treatment in the combined therapy group, 12 (36.4%) patients received single treatment and 8 (24.2%) patients received multiple treatments. Among the patients who received subsequent treatment in the monotherapy group, 27 (35.1%) patients received single treatment and 26 (33.8%) patients received multiple treatments (Additional file 1: Table S2). In general, there was no significant difference in the subsequent treatment received by patients between the two groups.

## Discussion

PVTT significantly reduces median survival (2.7–4 months) compared to patients without PVTT (10–24 months) [27], However, the optimal treatment for HCC with PVTT has not been established [28]. Systematic therapies (for example, VEGF inhibitors, tyrosine kinase inhibitors and immune checkpoint inhibitors) are recommended as the first-line treatment for HCC with PVTT [6, 7]. Indeed, the emergence of immune checkpoint inhibitors offers new strategies for the treatment of HCC. The most important breakthrough in this regard was the finding in the IMBrave150 trial [29] that atezolizumab in combination with bevacizumab achieved better overall and progression-free survival outcomes than sorafenib, and atezolizumab-bevacizumab has been adopted as first-line therapy [2, 30]. However, immune checkpoint inhibitors are not covered by the National Health Insurance in China, which would place a greater financial burden on patients than sorafenib or lenvatinib. Therefore, lenvatinib is currently the first choice in clinical practice, and our study provides new insight into the potential benefits of lenvatinib in combination with SBRT.

In the present study, median OS, PFS, IHPFS and ORR (based on mRECIST criteria) were significantly better in patients treated with lenvatinib in combination with SBRT than those in the lenvatinib monotherapy group. In addition, AEs in the combined therapy group were mostly manageable and the incidence was not statistically significantly different compared to the monotherapy group. Being satisfactory, lenvatinib plus SBRT reduced the risk of death by 63% (HR 0.37, 95% CI 0.23–0.60), and decreased the risk of tumor progression by 67% (HR 0.33, 95% CI 0.21–0.51), and lowered the risk of intrahepatic tumor progression by 71% (HR 0.29, 95% CI 0.18–0.45) compared with monotherapy. These survival differences may be associated with obtaining an improved treatment response (ORR 56.8% vs. 20.8%, *p* < 0.001). We also performed survival analysis in the SBRT plus lenvatinib group according to the treatment response status of patients, and the results suggested that OS was significantly higher in patients with a response than in those without a treatment response (*p* < 0.001). As the results of a phase 2 study of sorafenib in combination with radiation for advanced hepatocellular carcinoma [14] showed that radiation therapy may provide earlier tumor control than sorafenib and may reduce tumor load, which may allow sorafenib to exert a higher anti-tumor effect and ultimately contribute to survival by delaying tumor progress.

In our COX regression model, it was also demonstrated that the classification of PVTT was an independent factor affecting OS (*P* < 0.001). The classification of PVTT is closely related to the prognosis of HCC, and there are differences in survival outcomes with different treatments for different classification of PVTT [31]. Xi et al. [32] found that SBRT treatment for HCC with portal vein and/or inferior vena cava tumor thrombosis exhibited good local control rates with low toxicity. They also found that the combination of SBRT with sorafenib showed a trend toward prolonged median OS (16.6 months versus 10.9 months, *P* = 0.755), but no statistical difference was observed. In the STAH trial [33], it was found that TACE combined with sorafenib tended to prolong survival compared to sorafenib monotherapy for patients with Vp3 and Vp4 PVTT, but did not show a statistically significant difference. However, in another randomized trial of sorafenib combined with hepatic artery infusion chemotherapy (HAIC) versus sorafenib for hepatocellular carcinoma with major portal vein thrombosis (Vp3 and Vp4), the result showed the median OS and PFS were significantly prolonged in the combined treatment group [34]. Therefore, we divided the entire cohort of patients into Vp1-2 and Vp3-4 groups according to the degree of PVTT. In the subgroup analysis, the survival outcomes (OS, PFS and IHPFS) in the SBRT plus lenvatinib group were significantly longer than that in lenvatinib alone group. However, the proportion of Vp1 (7 cases) and Vp4 (10 cases) in our enrolled patients was small, so the results need to be interpreted with caution.

Notably, in consideration of the impact on liver function, all patients were treated with a sequential regimen (started oral lenvatinib treatment within 7 days after completion of the last SBRT) in this study. Li et al. [35] showed that sorafenib combined with radiotherapy produced schedule-dependent effects on HCC cells in vitro, which had important implications for the use of combination therapy in HCC patients. Therefore, the sequence of the RT and sorafenib combination is also important for treatment outcome. The study by Wild et al. [36] demonstrated that sequential sorafenib treatment after radiotherapy appears to be more effective for HCC. For lenvatinib combined with radiotherapy, a study of the pharmacokinetic and biodistribution effects of simultaneous or sequential lenvatinib with local liver irradiation in a freely moving rat model [37] demonstrated that a sequential regimen has a greater ability to maintain lenvatinib uptake compared to a simultaneous regimen, and that sequential regimens may be more impactful than concurrent regimens. However, there are fewer studies on the sequence of radiotherapy combined with TKIs, and further evidence is needed to elucidate the impact of the sequence between the two on treatment outcomes.

To our knowledge, this is the first clinical study comparing SBRT in combination with lenvatinib and lenvatinib alone for the treatment of HCC with PVTT. Recently, Yu et al. [38] conducted a retrospective clinical study of liver-directed radiotherapy (LRT) in combination with lenvatinib versus lenvatinib alone for the treatment of HCC with macroscopic tumor thrombosis and extrahepatic metastases. Their study showed that during a median follow-up period of 5.4 months (1.4–17.5 months), the combination treatment group had significantly better PFS (67.2% vs. 35.0% at 6 months, *p* = 0.008) and IHPFS (74.3% vs. 43.3% at 6 months, *p* = 0.008) were significantly better than in the monotherapy group, but there was no statistically significant difference in overall response rate (32.1% vs. 20.4%, *p* = 0.15) and OS rate (64.1% vs. 37.7% at 12 months, *p* = 0.06). This may be due to the fact that their study included more patients with extrahepatic metastases (LRT group: 42.9%, LRT plus lenvatinib group: 48.1%) and a shorter follow-up period. In contrast, patients with extrahepatic metastases were excluded in our study. The study of Zheng et al. [34] showed that in patients with extrahepatic dissemination, OS was longer in sorafenib monotherapy group than in sorafenib combined with HAIC group (HR 1.4; 95% CI 0.31–6.40). Therefore, the efficacy of treatment with radiotherapy combined with lenvatinib in patients presenting with extrahepatic metastases still need to be proven in further prospective clinical studies.

We acknowledge that there are some limitations to our study. Firstly, our study was retrospective, and although the baseline characteristics of both our patient groups were well balanced, our sample was still limited and could have led to various biases affecting survival outcomes. Subgroup analysis may have further reduced the sample size and therefore conclusions should be interpreted with caution. Secondly, we included patients with HCC with PVTT only and excluded patients with extrahepatic metastases, so it was not determined whether patients with both PVTT and extrahepatic metastases would benefit from SBRT in combination with lenvatinib therapy. Finally, in our study, the majority of patients were HBV (93.9%) positive, therefore, the efficacy of lenvatinib plus SBRT needs to be further confirmed in patients with HCC of other etiologies. Because of the above limitations, the results should therefore be interpreted with caution and validated in large sample randomized controlled trials.

## Conclusion

In conclusion, our study shows that the survival benefit of Lenvatinib in combination with SBRT for HCC with PVTT (either in patients with Vp1-2 or Vp3-4) is significantly better than Lenvatinib monotherapy and the adverse effects are well tolerated in combined treatment group.

## Supplementary Information

**Additional file 1. Table S1**. Baseline characteristics of the subgroup.** Table S2**. Subsequent treatment.

## Data Availability

The datasets used during the current study are available from the Corresponding author on reasonable request.
