# Supplementary material for: Lenvatinib with or without stereotactic body radiotherapy for hepatocellular carcinoma with portal vein tumor thrombosis: a retrospective study
Source: Radiat Oncol. 2023 Jun 12;18:101. doi: 10.1186/s13014-023-02270-z (PMC10259021; doi:10.1186/s13014-023-02270-z)
Supplement: Supplementary file 1 — Additional file 1. Table S1. Baseline characteristics of the subgroup. Table S2. Subsequent treatment. [file 13014_2023_2270_MOESM1_ESM.zip › Additional fle 1/Table S1.docx]

**Table S1**  Baseline characteristics of the subgroup

| Characteristics | Vp1/Vp2 groups | | P  value | Vp3/Vp4 groups | | P  value |
| --- | --- | --- | --- | --- | --- | --- |
|  | **SBRT+LEN group (n = 17)** | **LEN group (n = 34)** |  | **SBRT+LEN group (n = 20)** | **LEN group (n = 43)** |  |
| Gender, n (%)  Male  Female  Age, years, n (%)  ≥ 55 years  < 55 years  Etiology, n (%)  HBV  HCV  Child-Pugh classification, n (%)  A  B  ECOG PS score, n (%)  0  1  Number Of Tumors, n (%)  > 3  ≤ 3  Tumor size, n (%)  < 5 cm  ≥ 5 cm and < 10 cm  ≥ 10 cm  PVTT, n (%)  VP1  VP2  VP3  VP4  ALBI grade, n (%)  1  2  AFP, n (%)  > 200 ng/mL  ≤ 200 ng/mL  PLT, n (%)  ≥ 100 × 10^9^/L  < 100 × 10^9^/L  WBC, n (%)  ≥ 4 × 10^9^/L  < 4 × 10^9^/L  Previous local treatment, n (%)  Absence  Presence  TACE  Ablation  Argon–Helium cryosurgical | 16(94.1%)  1(5.9%)  11(64.7%)  6(35.3%)  15(88.2%)  2(11.8%)  16(94.1%)  1(5.9%)  10(58.8%)  7(41.2%)  7(41.2%)  10(58.8%)  3(17.6%)  11(64.7%)  3(17.6%)  1(5.9%)  16(94.1%)  -  -  6(35.3%)  11(64.7%)  5(29.4%)  12(70.6%)  13(76.5%)  4(23.5%)  13(76.5%)  4(23.5%)  14(82.4%)  2(11.8%)  1(5.9%)  0(0%) | 29(85.3%)  5(14.7%)  16(47.1%)  18(52.9%)  31(91.2%)  3(8.8%)  31(91.2%)  3(8.8%)  19(55.9%)  15(44.1%)  14(41.2%)  20(58.8%)  12(35.3%)  12(35.3%)  10(29.4%)  6(17.6%)  28(82.4%)  -  -  12(35.3%)  22(64.7%)  13(38.2%)  21(61.8%)  27(79.4%)  7(20.6%)  24(70.6%)  10(29.4%)  30(88.2%)  2(5.9%)  0(0%)  2(5.9%) | 0.650  0.234  1.000  1.000  0.842  1.000  0.136  0.472  1.000  0.756  1.000  0.912  0.673 | 16(80.0%)  4(20.0%)  10(50.0%)  10(50.0%)  19(95.0%)  1(5.0%)  15(75.0%)  5(25.0%)  7(35.0%)  13(65.0%)  10(50.0%)  10(50.0%)  4(20.0%)  8(40.0%)  8(40.0%)  -  -  17(85.0%)  3(15.0%)  7(35.0%)  13(65.0%)  13(65.0%)  7(35.0%)  15(75.0%)  5(25.0%)  12(60.0%)  8(40.0%)  20(100%)  0(0%)  0(0%)  0(0%) | 39(90.7%)  4(9.3%)  23(53.5%)  20(46.5%)  42(97.7%)  1(2.3%)  34(79.1%)  9(20.9%)  13(30.2%)  30(69.8%)  26(60.5%)  17(39.5%)  4(9.3%)  22(51.2%)  17(39.5%)  -  -  36(83.7%)  7(16.3%)  9(20.9%)  34(79.1%)  24(55.8%)  19(44.2%)  31(72.1%)  12(27.9%)  29(67.4%)  14(32.6%)  38(88.4%)  1(2.3%)  2(4.7%)  2(4.7%) | 0.435  0.796  0.538  0.971  0.705  0.435  0.452  1.000  0.232  0.491  0.809  0.564  0.169 |
| HBV, hepatic B virus; HCV, hepatic C virus; ECOG, Eastern Cooperative Oncology Group; PS, performance status; PVTT, Portal Vein Tumor Thrombosis; ALBI grade albumin-bilirubin grade; AFP, alpha-fetoprotein; PLT, platelet; WBC, white blood cell; TACE, transarterial chemoembolization; SBRT, Stereotactic Body Radiotherapy; LEN, lenvatinib. | | | | | | |
