# Supplementary material for: Lenvatinib with or without stereotactic body radiotherapy for hepatocellular carcinoma with portal vein tumor thrombosis: a retrospective study
Source: Radiat Oncol. 2023 Jun 12;18:101. doi: 10.1186/s13014-023-02270-z (PMC10259021; doi:10.1186/s13014-023-02270-z)
Supplement: Supplementary file 1 — Additional file 1. Table S1. Baseline characteristics of the subgroup. Table S2. Subsequent treatment. [file 13014_2023_2270_MOESM1_ESM.zip › Additional fle 1/Table S2.docx]

**Table S2** Subsequent treatment

| **Subsequent therapies** | **Groups** | | **P** |
| --- | --- | --- | --- |
|  | **SBRT + Len group (n = 33)** | **LEN group (n = 77)** |  |
| **Single treatment, n (%)**  TACE, n (%)  SBRT, n (%)  Argon–Helium cryosurgical, n (%)  Regorafenib, n (%)  PD-1, n (%)  **Multiple treatments**, **n (%)**  TACE + SBRT, n (%)  TACE + PD-1, n (%)  SBRT + PD-1, n (%)  Lenvatinib + PD-1, n (%)  TACE + Lenvatinib + PD-1, n (%)  **Best Supportive Care**, **n (%)** | **12 (36.4%)**  6 (18.2%)  4 (12.1%)  0 (0%)  1 (3.0%)  1 (3.0%)  **7 (21.2%)**  1 (3.0%)  2 (6.1%)  2 (6.1%)  1 (3.0%)  1 (3.0%)  **14 (42.4%)** | **25 (32.5%)**  15 (19.5%)  5 (6.5%)  3 (3.9%)  0 (0%)  2 (2.6%)  **24 (28.6%)**  4 (5.2%)  9 (11.7%)  7 (9.1%)  2 (2.6%)  2 (2.6%)  **28 (36.4%)** | **0.692**  0.874  0.448  0.553  0.300  1.000  **0.288**  0.399  0.579  0.879  1.000  1.000  **0.549** |
| TACE, Transarterial chemoembolization; SBRT, Stereotactic Body Radiotherapy; PD-1, programmed cell death-1 inhibitor; LEN, Lenvatinib. | | | |
